# The implementation of interprofessional education: a scoping review

**DOI:** 10.1007/s10459-022-10128-4

**Published:** 2022-06-10

**Authors:** Fiona Bogossian, Karen New, Kendall George, Nigel Barr, Natalie Dodd, Anita L. Hamilton, Gregory Nash, Nicole Masters, Fiona Pelly, Carol Reid, Rebekah Shakhovskoy, Jane Taylor

**Affiliations:** 1 School of Nursing, Midwifery and Paramedicine, University of the Sunshine Coast, Sippy Downs, Australia; 2 Sunshine Coast Health Institute, 6 Doherty Street, Birtinya, QLD 4575 Australia; 3 School of Health and Behavioural Sciences, University of the Sunshine Coast, Sippy Downs, Australia; 4 Consultant, Healthcare Evidence and Research, Brisbane, Australia; 5 School of Medicine and Dentistry, Griffith University, Sunshine Coast, Birtinya, Australia; 6 School of Preparation Pathways, University of the Sunshine Coast, Sippy Downs, Australia; 7 Sunshine Coast Hospital and Health Service, Birtinya, Australia

**Keywords:** Collaboration, Healthcare, Implementation, Interprofessional education, Interprofessional practice

## Abstract

**Introduction:**

Implementation of interprofessional education (IPE) is recognised as challenging, and well-designed programs can have differing levels of success depending on implementation quality. The aim of this review was to summarise the evidence for implementation of IPE, and identify challenges and key lessons to guide faculty in IPE implementation.

**Methods:**

Five stage scoping review of methodological characteristics, implementation components, challenges and key lessons in primary studies in IPE. Thematic analysis using a framework of micro (teaching), meso (institutional), and macro (systemic) level education factors was used to synthesise challenges and key lessons.

**Results:**

Twenty-seven primary studies were included in this review. Studies were predominantly descriptive in design and implementation components inconsistently reported. IPE was mostly integrated into curricula, optional, involved group learning, and used combinations of interactive and didactic approaches. Micro level implementation factors (socialisation issues, learning context, and faculty development), meso level implementation factors (leadership and resources, administrative processes), and macro level implementation factors (education system, government policies, social and cultural values) were extrapolated. Sustainability was identified as an additional factor in IPE implementation.

**Conclusion:**

Lack of complete detailed reporting limits evidence of IPE implementation, however, this review highlighted challenges and yielded key lessons to guide faculty in the implementation of IPE.

## Introduction

Interprofessional education (IPE) is key to the development of a collaborative practice-ready workforce. Interprofessional education promotes collaboration as participants review relationships between their professions, enhance mutual understanding and explore ways to combine their expertise towards improving delivery of service, patient safety and quality of care (World Health Organization, 2010a). The benefits of IPE and interprofessional collaborative practice (IPCP) are widely reported in the literature and include; role clarification, improved team functioning, enhanced conflict resolution and collaborative leadership, access to and coordination of care, appropriate use of specialist clinical resources, provision of optimal care, improved health care outcomes, reduced adverse consequences, reduced duplication of services, overcoming gaps in service provision, greater health worker productivity, inter-sectoral efficiency and community cohesion (Gilbert, 2018a, b; World Health Organization, 2010a).

The actions required to support IPE and IPCP are well described at a system level for health policy makers, but can be difficult concepts to explain, understand and implement (World Health Organization, 2010b). Implementation of IPE has been described as extremely challenging because of a deficit of quality methodological studies and staff resources (Lewy, 2010). However, examining implementation is important because a well-designed program can have differing levels of success depending on implementation quality (Gagnon et al., 2015). Unexpected outcomes, small effect sizes or inconsistent findings may not be related to the program design, but rather to poor program implementation (Caldwell et al., 2008).

As highlighted in the WHO Framework for Action on Interprofessional Education and Collaborative Practice, those responsible for implementing IPE should be competent and have expertise consistent with the nature of the planned IPE (World Health Organization, 2010b). However, many faculty (i.e., all health professional staff who have teaching roles) (Freeth et al., 2005) who have responsibility for implementing IPE need development to move beyond single professional approaches to implement learning experiences that are truly interprofessional (Ryland et al., 2017). While the importance of faculty development in IPE has been highlighted for many years (Freeth et al., 2005; Steinert, 2005) there is little evidence-based literature available to guide faculty development in IPE (Silver & Leslie, 2017), on the key knowledge and skills to implement IPE (Anderson et al., 2009). Consequently, the aim of this paper is to summarise the evidence for implementation of IPE and identify challenges and key lessons to guide faculty in IPE implementation.

To address the aim, scoping review methodology was chosen to enable a systematic search of the literature not restricted by study design (Cooper et al., 2019) and appropriate to explore the extent of evidence for IPE implementation. We did not register a review protocol. As a first step, we developed an operational definition of implementation as the activation of a specified set of planned and intentional activities of an intervention. The Interprofessional Curriculum Renewal Consortium Australia (2014) proposed a Teaching and Learning Decision Making which identifies “delivery” components and descriptors which were adopted as components and descriptors of implementation. These include curriculum (course, unit, activity), dimension (embedded/integrated or discrete/freestanding, mandatory or optional, implicit or explicit, individual or group learning, common or comparative learning, interactive or didactic learning), duration (hours, days, weeks, years), location (on campus, off campus), mode (face-to-face, online, blended), timing (synchronous, asynchronous), and teaching (individual, co-teaching, team teaching).

In addition to components of implementation, there are education factors which can influence the outcomes of IPE implementation and thus affect the health professional learner’s capacity to reach the goal of becoming an interprofessional collaborative practitioner. The Interprofessional Education for Collaborative Patient-Centred Practice (IECPCP) Framework proposed by D’amour and Oandasan (2005) provides a structure to categorise and understand education factors. The framework categorises education factors as micro or teaching factors (learner and educator professional and cultural beliefs and attitudes, learning context, faculty development), meso or institutional factors (leadership and resources, administrative processes), and macro or systemic factors (education system accreditation and institutional structures, social and cultural values that influence professional and cultural beliefs and attitudes). Combining the components of IPE implementation with the factors that influence outcomes was important to answer the research questions below.

## Methods

The method for this scoping review followed the stages recommended by Arksey and O’Malley (2005) and Levac et al. (2010). The PRISMA Extension for Scoping Reviews (PRISMA-ScR) (Tricco et al., 2018) was selected to guide the reporting of this review.

### Identifying the research question

The research questions for this review were: (1) What are the methodological characteristics and implementation components reported in primary IPE studies? and (2) What are the challenges and key lessons for faculty to consider when implementing IPE?

### Identifying relevant studies

A Senior Librarian managed the search of Scopus, Web of Science, ERIC (Education Resources Information Center), PsycInfo, CINAHL (EBSCOHost) and the Cochrane Library databases in collaboration with the project team. The key words ‘interprofessional education’, ‘interprofessional learning’ and ‘interprofessional collaboration’ were searched in title, abstract and keywords of articles. Filters applied to searches included human participants, English language, and publication date between 2010 and 2019. The full electronic preliminary search strategy is provided below.

**Table:** 

Database	SCOPUS	Web of Science	ERIC	PsychInfo	CINAHL	Cochrane Reviews
Date coverage	2010 to 2019					
Date of search	16/05/2019					
Limits	Language: English Document type: Original research and Review Subject areas: health topics					
Search query (keywords)	“interprofessional education” OR “inter-professional education” OR “interdisciplinary education” OR “inter-disciplinary education” OR “interprofessional learning” OR “inter-professional learning					
Number of hits	393	150	5	53	4	2

The search was subsequently refined to terms ‘interprofessional assessment’ OR ‘interprofessional design’ OR ‘interprofessional implementation’ OR ‘interprofessional evaluation’. Peer-reviewed original research and reviews, regardless of methodological approach were eligible for inclusion. Theses and grey literature (technical reports, government papers, conference proceedings) were excluded.

### Study selection

The results were collated in Endnote 7 (2013) by the Senior Librarian, who removed duplicates which, in turn, were checked by one team member (KN). Then two members (KN and FB) screened titles and abstracts independently for potential eligibility which included reporting on IPE design, implementation, assessment, and/or evaluation involving learners from two or more professions, where at least one of the professions was from a list of 25 regulated and self-regulated professional groups in Australia. Disciplines included were Chinese medicine, chiropractic, counselling, dietetics, dentistry, exercise physiology, Indigenous or First Nations’ health, medical imaging, medicine, midwifery, nursing, nutrition, occupational therapy, optometry, osteopathy, paramedicine, pastoral care, pharmacy, physiotherapy, podiatry, psychology, public health, physician assistant, social work, and speech therapy. Screening results were compared, and non-agreement resolved though discussion.

Following the retrieval of the full texts for papers identified as potentially eligible, six pairs of team members (NB-KG; AH-NM; GN-JT; FP-RS; CR-ND; FB-KN) were assigned a portion of the papers to determine the focus of the paper and which component/s of IPE were addressed; design, implementation, assessment and/or evaluation. One of two team members (KN, FB) arbitrated disagreements or uncertainty between paired team members. The papers deemed eligible were organised under each of the four domains by one team member (KN), and papers identified for the implementation domain that reported on primary studies progressed to the data charting stage for this review.

### Charting the data

For this stage, three team members (NB, KG, FB) were assigned to systematically review each paper identified for this domain, confirm its inclusion, and chart implementation data based on the components previously identified. Data not explicitly reported were extrapolated where possible. Each paper was then re-assessed by FB and KN, ensuring components that contributed to IPE implementation were captured, and key lessons and challenges reported by the authors of the individual papers were charted.

### Collating, summarising and reporting the results

Data relating to the components of implementation were mapped and summarised (Chelimsky, 1989; Stemler, 2001; Tashakkori & Teddlie, 2010) and data relating to challenges and key lessons were compared for points of consistency through an interactive process of describing, classifying and connecting information. To summarise the challenges and key lessons for faculty implementing IPE, thematic analysis of the textual data using the education factors of the IPECPCP framework (D’amour & Oandasan, 2005) was undertaken by one author (FB). The analysis was then independently checked by one other researcher (KN) and then collaboratively summarised.

## Results

**Fig. 1:**
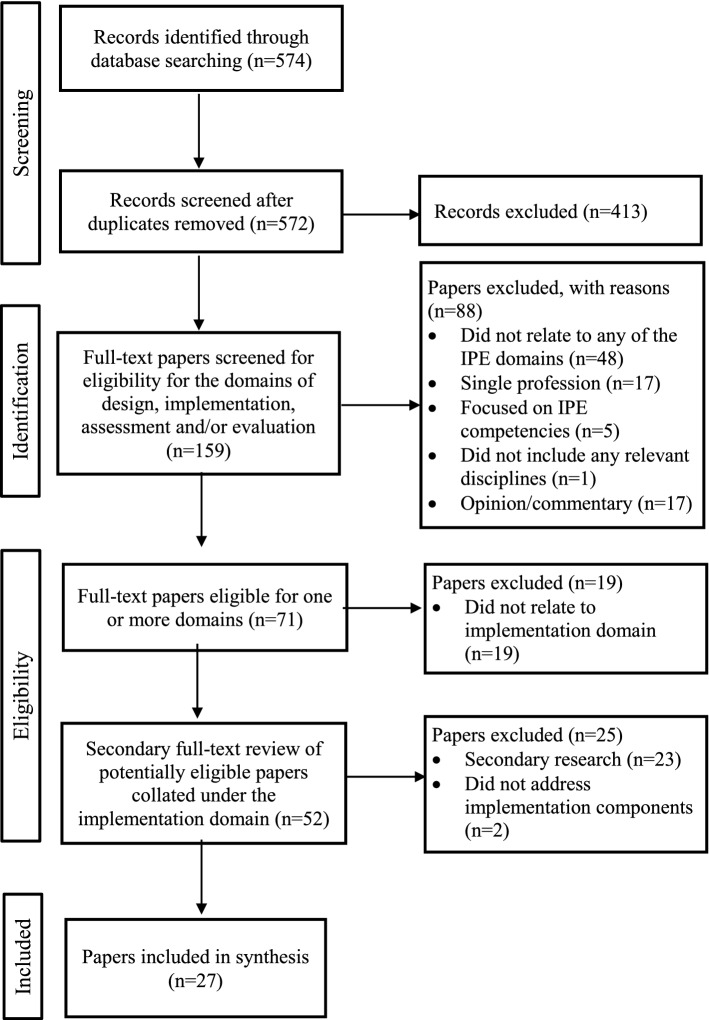
PRISMA flow diagram for the paper selection process

A total of 27 papers met the inclusion criteria and were confirmed for inclusion in this review (Fig. 1).

### Characteristics of included studies

The characteristics of included studies are presented in Table 1. Studies were undertaken in the United States of America (USA) (n = 15), Canada (n = 6), United Kingdom (UK) (n = 3), Australia & New Zealand (Lapkin et al., 2012), Belgium (n = 1), and Sweden (n = 1).

**Table 1:** Methodological characteristics and implementation components of included studies

Author, year Country	Methodological characteristics	Implementation components							
		Curriculum	Dimension		Duration	Location	Mode	Timing	Teaching
Acquavita et al. (2014) USA	*Mixed-methods* Described the administration of the RIPLS questionnaire to 29 students from five health sciences schools, including law, and the findings from semi-structured interviews	Informal, varied, some courses	Discrete or integrated		NR	On campus & Off campus NSS	Face-to-face	NR	NR
			Mandatory or optional	Both NSS					
			Implicit or explicit						
			Individual or group						
			Common or comparative						
			Interactive or didactic						
Cusack and O’Donoghue (2012) UK	*Cross-sectional study* Described the evaluation of an IPE Problem Based Learning (PBL) module using a questionnaire with quantitative and qualitative components by health science disciplines	Module	Discrete or integrated		12-week semester	On campus	Blended	NR	Individual
			Mandatory or optional	Both					
			Implicit or explicit						
			Individual or group						
			Common or comparative						
			Interactive or didactic	Both					
Dando et al. (2012) UK	*Case report* Described the evaluation of an interprofessional practice placement (IPP) within a palliative care unit using four methods of evaluation and triangulation	Activity—practice placement	Discrete or integrated		Three-week blocks in six rotations	On campus & Off campus	Face-to-face	NR	Team
			Mandatory or optional	Optional					
			Implicit or explicit						
			Individual or group						
			Common or comparative						
			Interactive or didactic						
Dean et al. (2014) Canada	*Case report* Described the development of an IPE clinical placement for health professional students and lessons learned over 6 years	Activity—clinical placement	Discrete or integrated		Two to 12 weeks discipline dependent	Off campus	Face-to-face	NR	Individual and team
			Mandatory or optional	Mandatory components					
			Implicit or explicit						
			Individual or group						
			Common or comparative						
			Interactive or didactic						
Deutschlander et al. (2012) Canada	*Case report* Presented a pre-licensure pilot interprofessional (IP) intervention and discussed strategies and challenges of implementing IPE interventions with students from different disciplines	Activities—clinical placement	Discrete or integrated	Integrated	One semester	Off campus	Blended	Synchronous NSS	Co-teaching
			Mandatory or optional	Optional					
			Implicit or explicit						
			Individual or group	Both					
			Common or comparative						
			Interactive or didactic						
Di Prospero and Bhimji-Hewitt (2011) Canada	*Cohort study* Presented the analysis of a cohort of faculty discussions that occurred during debrief sessions on how to better foster collaboration and efficiently manage student participation	Course	Discrete or integrated		One semester	On campus	Face-to-face	Synchronous NSS	Team
			Mandatory or optional						
			Implicit or explicit						
			Individual or group						
			Common or comparative						
			Interactive or didactic	Interactive					
Djukic et al. (2012) USA	*Cohort study* Described the plan to measure teamwork and collaboration knowledge, skills and attitudes longitudinally within a single cohort of medical and nursing students at 0, 5 and 12 months	Course	Discrete or integrated	Integrated	One year	NR	Online	Asynchronous	Individual
			Mandatory or optional	Mandatory					
			Implicit or explicit						
			Individual or group	Both					
			Common or comparative						
			Interactive or didactic	Both					
Evans et al. (2011) USA	*Case report* Described how teams from different institutions, consisting of at least three faculty members from different health professions, attended a workshop that facilitated development of proposals for educational initiatives to advance IPE	Varied—courses and activities	Discrete or integrated		Varied	NR	Blended	Synchronous & Asynchronous	Varied NSS
			Mandatory or optional	Both					
			Implicit or explicit						
			Individual or group						
			Common or comparative						
			Interactive or didactic	Didactic					
Galbraith et al. (2014) USA	*Cross-sectional study* Described the development and implementation of the interprofessional death notification simulation involving students and standardised patients	Activity integrated into two courses in social work and nursing	Discrete or integrated		45 min	On campus	Blended	Synchronous	Team
			Mandatory or optional						
			Implicit or explicit						
			Individual or group						
			Common or comparative						
			Interactive or didactic	Interactive					
Grant et al. (2011) Canada	*Case report* Described the results of an IPE initiative between dental hygiene and nursing professions to prepare students for collaboration practice	Activity	Discrete or integrated		Two-hour session (oral health); NR. (blood pressure)	On campus	NR	Synchronous NSS	Co-teaching
			Mandatory or optional						
			Implicit or explicit						
			Individual or group	Group					
			Common or comparative						
			Interactive or didactic	Interactive					
Grymonpre (2016) Canada	*Case report* Described strategies that fostered interprofessional faculty development within a university IPE initiative and evaluations of each strategy	Course	Discrete or integrated		One week	On campus	Face-to-face	Synchronous NSS	NR
			Mandatory or optional						
			Implicit or explicit						
			Individual or group	Both					
			Common or comparative						
			Interactive or didactic	Both					
Gummesson et al. (2018) Sweden	*Case report* Presented a conceptual idea of using clinical reasoning as a framework for IPL	Module	Discrete or integrated	Discrete	NR	NA	Blended	Synchronous & Asynchronous	Co-teaching
			Mandatory or optional	Optional					
			Implicit or explicit						
			Individual or group						
			Common or comparative						
			Interactive or didactic						
Kaplan et al. (2015) USA	*Case report* Described the incorporation of midwifery students into 2 courses previously offered only to medical students, including an evaluation component	Two courses	Discrete or integrated		NR	On campus	Face-to-face (Course 1); blended (Course 2)	NR	Individual
			Mandatory or optional	Course 1 Mandatory for nurses & midwives, Optional for medical Course 2 only some sessions mandatory for nurses & midwives					
			Implicit or explicit						
			Individual or group	Course 2 group					
			Common or comparative						
			Interactive or didactic	Course 1 Didactic Course 2 interactive					
Krystallidou et al. (2018) Belgium	*Case report* Described a training initiative between medical and interpreter students to improve collaboration and communication between the two disciplines in preparation for interpreter-mediated communication with patients	Activity	Discrete or integrated	Integrated for medical students, discrete for interpreter students	One plenary lecture + 30 min practice session for medical students; 90 min interpreter students NSS	On campus	Face-to-face	Synchronous NSS	Co-teaching
			Mandatory or optional						
			Implicit or explicit						
			Individual or group						
			Common or comparative						
			Interactive or didactic						
Lapkin et al. (2012) Australia & New Zealand	*Cross-sectional study* Described the results of a descriptive cross-sectional survey undertaken to ascertain information on the extent and scope of IPE use in Australian and New Zealand nursing, pharmacy and medical programs to teach medication safety and identify barriers and facilitators	Lectures (16%), Tutorials (16%), Clinical placements (15%) and Simulation (12%)	Discrete or integrated		NR	On campus & Off campus	Face-to-face (46%), online (22%) and combined blended & distance (28%)	NR	NR
			Mandatory or optional	Mandatory (69%), Optional (12%) with academic credit, extra-curricular with no academic credit (19%)					
			Implicit or explicit						
			Individual or group						
			Common or comparative						
			Interactive or didactic	Both					
Masters et al. (2013) USA	*Case report* Described development of a multidisciplinary curriculum model for providing IPL experiences for students via the patient simulation centre at a university	Activities	Discrete or integrated	Integrated	Final year (seven content areas offered in five phases) NSS	On campus	Face-to-face	Synchronous NSS	Team
			Mandatory or optional						
			Implicit or explicit						
			Individual or group						
			Common or comparative						
			Interactive or didactic	Both					
Mendel et al. (2015) USA	*Pilot study* Described a pilot study of podiatric medical students and student registered nurse anaesthetist’s IPE simulation experience	Activity	Discrete or integrated	Discrete	Two-hour	Simulation laboratory NSS	Face-to-face	Synchronous NSS	Co-teaching
			Mandatory or optional	Optional					
			Implicit or explicit						
			Individual or group	Group					
			Common or comparative						
			Interactive or didactic						
Michalec et al. (2017) USA	*Case report* Explored attitudes and perceptions of students from 6 health disciplines and potential barriers and facilitators to students’ engagement	Course	Discrete or integrated	Discrete	Two years	On campus & Off campus	Face-to-face	NR	Team
			Mandatory or optional	Mandatory					
			Implicit or explicit						
			Individual or group	Group					
			Common or comparative						
			Interactive or didactic						
Packard et al. (2018) USA	*Case report* Discussed the development of an ‘IPE passport’ as an innovative way to address learners needs across seven health professions	University-wide course with activities	Discrete or integrated	Both	Over the course of the respective degrees	On campus & Off campus	Online	Synchronous & Asynchronous	NR
			Mandatory or optional	Mandatory					
			Implicit or explicit						
			Individual or group						
			Common or comparative						
			Interactive or didactic						
Reis et al. (2015) USA	*Case report with Pre and* *Post-Test* Described the experience of IPE using a Virtual Community Clinic Learning Environment (VCCLE) for nurse-midwifery and medical students	Virtual Activities + online learning module, asynchronous discussion board	Discrete or integrated		Four—six hours	Online	Online	Asynchronous	NR
			Mandatory or optional	Mandatory					
			Implicit or explicit						
			Individual or group						
			Common or comparative						
			Interactive or didactic						
Shaw-Battista et al. (2015) USA	*Case report* Described childbirth curriculum development and simulation implementation within the nurse-midwifery education program	Modules	Discrete or integrated		NR	On campus & Off campus	Blended	Synchronous NSS	Co-teaching
			Mandatory or optional						
			Implicit or explicit						
			Individual or group						
			Common or comparative						
			Interactive or didactic	Both					
Tartavoulle et al. (2016) USA	*Case report with Pre and* *Post-Test* Described the use of the IDEA Framework to design learning activities and assess competency related to roles and responsibilities in health professions students	Introductory course	Discrete or integrated		Two credit hours + four case-based small group sessions Total duration unclear	On campus	Face-to-face	Synchronous NSS	NR
			Mandatory or optional	Optional					
			Implicit or explicit						
			Individual or group	Group					
			Common or comparative						
			Interactive or didactic	Interactive and didactic					
Topping (2015) USA	*Case report* Described a short course and student evaluation of IPE and cultural competency activities	Course	Discrete or integrated		15 h of total instruction	Online	Online	NR	Co-teaching
			Mandatory or optional	Optional					
			Implicit or explicit						
			Individual or group	Group					
			Common or comparative						
			Interactive or didactic						
Vanderzalm et al. (2013) Canada	*Case report with Pre and* *Post-Test* Described the development, implementation and evaluation of an innovative model for IPC—the IP clinical learning unit	Activities	Discrete or integrated		NR	Off campus	Blended	NR	NR
			Mandatory or optional						
			Implicit or explicit						
			Individual or group						
			Common or comparative						
			Interactive or didactic						
VanKuiken et al. (2016) USA	*Case report* Described a multifaceted IPE program that engaged students from a variety of disciplines and experience levels	Course, activities	Discrete or integrated	Integrated	Four-10 h. NSS	On campus	Face-to-face or blended	NR	Team
			Mandatory or optional						
			Implicit or explicit						
			Individual or group	Group					
			Common or comparative						
			Interactive or didactic						
Watts et al. (2014) USA	*Case report* Described the process, simulation scenarios and set up	Activity	Discrete or integrated	Integrated	90 min	On campus	Face-to face	Asynchronous	Team
			Mandatory or optional	Optional for some students and mandatory for others					
			Implicit or explicit						
			Individual or group						
			Common or comparative						
			Interactive or didactic						
Welsh 2012 UK	*Case report* Described qualitative finding: many participants do not always understand the terminology used from one acute care and treatment (ACT) course	Course	Discrete or integrated		One day	NR	Face-to-face	NR	Team
			Mandatory or optional						
			Implicit or explicit						
			Individual or group						
			Common or comparative						
			Interactive or didactic						

*NR* not reported, *NSS* not specifically stated/inferred, *NA* not applicable

Most of the studies were descriptive in design and 17 were classified as case reports, three cross sectional, and one mixed methods study. Analytical observational designs included two cohort studies, one pilot study and pre and post-test design elements were included in three case reports. There were no experimental or quasi-experimental studies.

Included studies largely reported directly on the implementation of IPE programs or activities directed to health profession learners. Four studies reported indirectly on components of program implementation by drawing on analysis of faculty discussions during debrief sessions (Di Prospero & Shimji-Hewitt, 2011), facilitation of faculty members from different institutions and professions in development of IPE (Evans et al., 2011), strategies that fostered IPE faculty development (Grymonpre, 2016), and a survey to determine the extent, scope, barriers and facilitators to IPE use (Lapkin et al., 2012).

### Implementation components of included studies

The implementation components of IPE were variously reported (or could be inferred) across the included studies, with timing and teaching components the least frequently reported. Curriculum components were universally reported and varied from a university-wide course (Packard et al., 2018) to courses, modules or activities—some of which occurred during placement.

The dimension components were inconsistently reported. Only eight studies reported whether the activities were discrete or integrated into curricula, with two studies identified as having discrete IPE, four as having integrated IPE, and two studies having both.

More frequently reported was whether IPE was implemented as mandatory or optional, with five studies reporting mandatory, six studies reporting optional, a further six studies reporting both possibilities for all learner groups, and two studies reporting that IPE was mandatory for some learner groups and optional for others. In their cross-sectional survey of 31 Universities, Lapkin and colleagues (2012) reported that 69% of programs reported mandatory IPE and made the distinction between optional IPE with academic credit (12%) and extracurricular without academic credit (19%).

None of the included studies reported nor could it be inferred as to whether the IPE was implemented implicitly for learners or made explicit during activities. Likewise, none of the studies reported on common or comparative learning across the professions. Group learning was reported in seven studies, and a combination of individual and group learning was reported in three studies. No studies reported individual learning. Implementation of IPE using interactive methods were reported in three studies and didactic methods in one study. Six studies reported using both methods.

Most of the studies reported on implementation duration, which varied from short 45-minute sessions to week/s, semester or year-long and whole of programme durations. However, six studies did not report on duration, nor could this be inferred (Table 1). The location of IPE implementation was on campus in 11 studies, off campus in three studies and both on and off campus in six studies.

The mode of IPE implementation was predominantly face-to-face (n = 12), or blended (n = 7). Two studies reported using either face-to-face or blended modes and four using online mode only. Lapkin and colleagues (2012) reported face-to-face (46%), blended and distance (28%), and online (22%) in their cross-sectional survey.

The timing of IPE implementation was identified as synchronous in nine studies, asynchronous in three studies or as both in three studies. The remaining studies did not report on timing of implementation.

IPE was implemented using individual teaching in three studies, co-teaching in seven studies and team teaching in a further eight studies. When co-teaching and team teaching were implemented, teachers’ professions were representative of the learner professions, with medicine, nursing and therapies being the most frequently reported. Two studies reported varied teaching implementation.

### Challenges and key lessons in included studies

Interprofessional education implementation challenges and key lessons were extracted from the text of the included studies and mapped against the themes of micro (teaching), meso (institutional) and macro (systemic) level factors (D’amour & Oandasan, 2005). Factors that fell outside this framework were identified as ‘other’.

#### Micro level

Three teaching factors were identified in the included studies that could affect the learners’ capacity to become a competent collaborative practitioner namely, socialisation issues, learning context, and faculty development.

*Socialisation issues* (professional and cultural beliefs and attitudes that develop among health professionals) of learners and educators were identified as challenges in eight studies. Being unreceptive to learning from other professionals (Acquavita et al., 2014), professional silos (Packard et al., 2018), learners’ perceptions of unequal status and role identification (Dando et al., 2012), negative stereotyping and misperceptions (Acquavita et al., 2014; Michalec et al., 2017), insufficient professional identity formation in learners (Michalec et al., 2017) and professional lack of awareness of similarities and differences in thinking (Gummesson et al., 2018) were identified as professional culture challenges. Additionally, there was scepticism and lack of buy in to IPE from others including learners (Di Prospero & Shimji-Hewitt, 2011) and learner resistance (Lapkin et al., 2012) which challenged learner engagement with IPE.

Key lessons—*Socialisation issues*.

- There is a need to acknowledge and address socialisation issues: hierarchical barriers and stereotyping (Di Prospero & Shimji-Hewitt, 2011; Grymonpre, 2016), differences across disciplines (Evans, et al., 2011), status differences (Deutschlander et al., 2012), and convey that IPE is equally as important as clinical topics (Djukic et al., 2012).

*Learning context* which reflects the ‘who, what, where and when’ of IPE (D’amour & Oandasan, 2005) also presented challenges to implementation. In terms of ‘who’ is involved in IPE, learner considerations were variability in student numbers and mix from different professions (Dando et al., 2012), disparity in health professions students clinical experiences (Kaplan et al., 2015) and learning needs (VanKuiken et al., 2016). Faculty challenges included the variability in appointment of preceptors across professions (Dean et al., 2014).

Key lessons—*Learning context*.

Key lessons—*Learning context - *Who.

- Managing group diversity is important (Welsh, 2012) however the focus should be on a cohesive approach and developing understanding between disciplines when common elements are being taught (Masters et al., 2013).
- Learners should be included more in, or perhaps lead the debrief sessions to promote their engagement in the interprofessional team approach (Lapkin et al., 2012).
- Interprofessional educator teams and collaborative practice teams are needed in both classroom and clinical settings (Acquavita et al., 2014) and should role model interprofessional team collaboration and communication (Shaw-Battista et al., 2015).
- Interprofessional faculty team members must respect and accommodate different levels of confidence, experience and enthusiasm for teaching and mentoring learners from different professions (Dean et al., 2014).
- Interprofessional mentoring in existing placement courses is highly successful (Deutschlander et al., 2012) and involving the patient as mentor can enhance IPE experience (Michalec et al., 2017).

With respect to the ‘what’ of IPE challenges, faculty considerations included shifting the focus of learning from knowledge and tasks to IPE competencies such as teamwork and communication (Lapkin et al., 2012) while meeting each discipline’s IPE requirements (Packard et al., 2018). During implementation of ‘what’ is being taught, not all activities were as collaborative as intended (Packard et al., 2018) and the gap between what was planned by faculty and what was experienced by learners may require increased attention to factors that impede learner engagement.

Key lessons—*Learning context - *What.

- Small group learning enables the development of interprofessional collaborative practice competencies in communication, teamwork, problem solving, independent responsibility for learning, sharing information and respect for others (Cusack & O’Donoghue, 2012), and is more effective than large group discussion (Tartavoulle et al., 2016).
- Adult learning principles should be applied to integrate practice experiences, knowledge acquisition, reflection (Deutschlander et al., 2012) in interprofessional discussion (Kaplan et al., 2015) and to link small group sessions to relevant course content and professional clinical practice (Di Prospero & Shimji-Hewitt, 2011).
- Those in the early stages of IPE, should consider innovative pedagogies (such as IPE Passports) complemented by clear strategy for successful implementation (Packard et al., 2018).
- Authentic scenarios (Krystallidou et al., 2018) and case studies of patients which highlight different treatment needs, discipline involvement, collaborative interaction and reflection (Vanderzalm et al., 2013) that have flexibility in scenario timelines to reflect clinical decision making (Watts et al., 2014) should be used but may need to be modified during implementation depending on disciplines, experience and learning objectives (Shaw-Battista et al., 2015).
- Formal education and structured activities in IPE are necessary (Tartavoulle et al., 2016) and both formal and informal opportunities should be implemented to assist direct learner engagement (Michalec et al., 2017).
- Learners with little clinical experience will require knowledge of interprofessional collaborative practice, whereas learners with clinical experience will require more interprofessional skills development (VanKuiken et al., 2016).
- Learners need to understand each other’s professional language in order to improve communication (Grant et al., 2011) and should be encouraged to consider how they might use each other’s professional skills (Grymonpre, 2016).

Challenges which reflected ‘where’ in terms of learning context did not specifically relate to whether the learning took place in the academic institution or the hospital environment, in the classroom, on clinical placement or in the virtual environment. However, the challenges included the implementation of authentic experiences such as real-time, multi-patient simulations involving multiple professions (Watts et al., 2014), the provision of physical space for teamwork, and a respectful learning and working environment (Dean et al., 2014).

Key lessons—*Learning context -* Where.

- Authentic, multi-professional learning environments (whether in clinical settings, training wards or in realistic simulation environments) are critical to enhance the preparation of learners in roles (Galbraith et al., 2014) and provide opportunities for reflection and debrief (Cusack & O’Donoghue, 2012).
- Asynchronous, modular, web-based, on-line learning can be beneficial (Lapkin et al., 2012) and overcome the lack of physical space (Djukic et al., 2012) but they may not support opportunities for interprofessional conversation and exchange of ideas (Kaplan et al., 2015) or lend themselves to shared experiences.

Curriculum implementation considerations illustrated the ‘when’ of IPE and included the impact of elective (Dean et al., 2014) and optional activities on participation (Deutschlander et al., 2012; Shaw-Battista et al., 2015) and building new ideas and concepts into curricula full of uni-professional content (Reis et al., 2015).

Key lessons— *Learning context - *When.

- Interprofessional education requires a unique type of curriculum, with defined curriculum structures that facilitate and promote interaction and group learning between disciplines (Acquavita et al., 2014), and provide for demonstrable evidence of collaboration with importance placed on the value of learning with and from each other (Cusack & O’Donoghue, 2012).
- An induction programme (Dando et al., 2012) or orientation sessions should be implemented for learners to understand goals, activities, and participation (Deutschlander et al., 2012), develop a set of rules around group role expectations (Di Prospero & Shimji-Hewitt, 2011), address the roles of each profession (Kaplan et al., 2015), and the impact of interprofessional collaborative practice on health care system and patient outcomes (Di Prospero & Shimji-Hewitt, 2011).
- Rich interprofessional learning experiences require pre-brief discussion to create a supportive learning environment (Shaw-Battista et al., 2015) and facilitate discussion in the classroom (Kaplan et al., 2015).
- The timing of interprofessional experiences in the curricula needs careful consideration about whether learners have early or later exposure or, whether exposure is early and continuous (Lapkin et al., 2012).
- Mandatory intra-curricular interprofessional experiences will support attendance and group participation (Cusack & O’Donoghue, 2012) while optional or elective extra-curricular experiences are self-selected and participants are motivated and interested (Tartavoulle et al., 2016).
- Curriculum implementation should address how to support students who go off track, manage end of semester reporting, non-attendance and, unprofessional behaviour (Packard et al., 2018).

*Faculty development* represents the final micro factors. It addresses the need to learn how to facilitate IPE and to recognise ones’ own professional beliefs and attitudes about collaboration (D’amour & Oandasan, 2005) and is also revealed as challenges in the included studies. To optimise the success of IPE, expert facilitation and facilitator support and training are required (Di Prospero & Shimji-Hewitt, 2011) to cultivate buy-in and create a critical mass of faculty who understand IPE. However, dedicated IPE faculty with formal training is not a common practice (Dean et al., 2014). Lack of faculty time, sufficient interested faculty (C Evans, H et al., 2011), lack of faculty flexibility and, willingness to work with each other (Grant et al., 2011) were identified as challenges in the included studies.

Key lessons—*Faculty development*.

- Faculty development and competence in IPE is critical to successful implementation (Acquavita et al., 2014) and faculty from each profession need to be involved in the team for implementation (Kaplan et al., 2015).
- A theoretical framework should be used to guide desired learning outcomes for faculty development (Grymonpre, 2016).
- Faculty development requires formalised training to enable faculty to develop a shared understanding of IPE, be prepared to address issues faced including managing tensions, hierarchal barriers and cultural tensions and be attuned to the dynamics of interprofessional learning (Di Prospero & Shimji-Hewitt, 2011).
- Ongoing faculty development (Shaw-Battista et al., 2015) using regular team meetings, student free time (Dean et al., 2014) and joint faculty debrief sessions are valuable to facilitate faculty learning, team development (Grant et al., 2011) and role development (Di Prospero & Shimji-Hewitt, 2011).
- Interprofessional facilitation guides (with key points for discussion and debrief) are needed to support new faculty (Di Prospero & Shimji-Hewitt, 2011).
- Faculty need role models (Acquavita et al., 2014) who demonstrate collaboration by modelling interprofessional behaviour, respectful cooperation and valuing of input from others (Masters et al., 2013).

#### Meso-level

Institutional factors of leadership and resources as well as administrative processes impact the implementation of IPE regardless of whether it is conducted in the academic or hospital environment (D’amour & Oandasan, 2005).

*Leadership and resources* refer to administrators having the power to advance IPE by providing resources and champions to support the vision (D’amour & Oandasan, 2005), and was described by one study as balancing buy in with infrastructure to ensure quality IPE experiences (Packard et al., 2018). Leadership included lack of institutional (Lapkin et al., 2012) and higher-level support (Packard et al., 2018) and was linked to the engagement of clinical leaders and management (Vanderzalm et al., 2013) and of other faculty who were not leading the IPE activities (Packard et al., 2018).

Resources were highlighted in the included studies and challenges related to limitations (Djukic et al., 2012), availability (Grant et al., 2011), the appropriateness of teaching and learning resources (Lapkin et al., 2012), and funding (Lapkin et al., 2012; Reis et al., 2015) were recognised. Implementation of IPE activities was identified as having impact on resources. Placements were resource intensive (Dando et al., 2012), multi-patient simulation required a large number of staff (Watts et al., 2014) and large scale technology mediated IPE required significant financial resources and staff with expertise in educational technology and instructional design (Djukic et al., 2012). Activities including curriculum revision, planning, and implementing IPE also required staff time (Shaw-Battista et al., 2015). In the environment of a large health care facility, site facilitation, coordination, and management of resources (Vanderzalm et al., 2013) also posed challenges.

Key lessons—*Leadership and resources*.

- To support IPE implementation strong committed leadership (Dean et al., 2014) with a clear strategy, thoughtful approach, and measured responses to potential challenges are needed (Packard et al., 2018).
- Transformational leadership which engages both faculty and staff as core champions and leverages early adopters is important to successful implementation (Packard et al., 2018).
- Pre-implementation steps include creating a shared vision, developing resources, identifying clear roles and securing financial support (VanKuiken et al., 2016).

*Administrative processes* refer to methods for implementing initiatives including logistical decisions and financial incentives (D’amour & Oandasan, 2005). In terms of logistics, timing was identified as a challenge (Acquavita et al., 2014) in response to clinical placement variation (Dean et al., 2014) as a result of diversity in curricula timing where content and learning experiences occur (Masters et al., 2013) and a lack of time for interaction (Reis et al., 2015). This was distinct from issues with timetabling (Cusack & O’Donoghue, 2012; Grant et al., 2011) and scheduling (Masters et al., 2013; Packard et al., 2018) relating to students being from different professions (Dando et al., 2012) with different schedules (C Evans, H et al., 2011) and at varied levels (Shaw-Battista et al., 2015) merging curricula with fixed schedules (Kaplan et al., 2015) and arranging meetings with team members and mentors (Michalec et al., 2017; VanKuiken et al., 2016).

Location also posed challenges in blending students who are full-time, on campus with those working clinically and studying part-time (VanKuiken et al., 2016) and in relation to the coordination of activities in different locations and community logistics (C Evans, H et al., 2011). Providing participation experiences for large numbers of students (Galbraith et al., 2014) or when student numbers in one profession exceeds all other groups (VanKuiken et al., 2016), was also challenging. Technology issues were also identified as costly and time consuming in one study (Reis et al., 2015). None of the included studies reported on financial incentives.

Key lessons—*Administrative processes*.

- Logistical challenges are threats to IPE implementation and managing these requires regular meetings of directors, faculty, supporting staff and instructional designers (Packard et al., 2018).
- Engaging administrators is critical (VanKuiken et al., 2016) to successful implementation.

#### Macro level

The macro or *systemic factors* which can influence the implementation of IPE are threefold. Firstly, the education system which includes accreditation and institutional structures, secondly government policies on education, health and social services and finally social and cultural values that influence professional beliefs and attitudes. Education system factors identified in the included studies were institutional policies (Lapkin et al., 2012), rigid program requirements (Acquavita et al., 2014), burdensome institutional approval for new courses (Deutschlander et al., 2012), obtaining course approval across academic units and allocating course credits (C Evans, H et al., 2011). The downstream impact of government policies is reflected in different accreditation standards (Packard et al., 2018), legislative requirements (Lapkin et al., 2012), and regulatory and credentialing requirements (VanKuiken et al., 2016) for each profession. Social and cultural factors included hidden power structures (Grymonpre, 2016) and role perceptions. Clinical leaders and managers expressed that interprofessional roles and functions fell outside busy front-line positions (Vanderzalm et al., 2013).

Key lessons—*systemic factors*.

- Institutional policies for academic credit for participation in IPE initiatives, should be established and embedded in curricula (Grant et al., 2011) to support effective implementation of IPE.
- Enhancement opportunities that do not require onerous institutional, large scale faculty review and approval (Deutschlander et al., 2012) should be considered as an IPE implementation approach.
- Partnerships within and between academia and health care delivery organisations are important (Grymonpre, 2016) to implementation of IPE.
- High level institutional support (Cusack & O’Donoghue, 2012; Djukic et al., 2012) that includes strong collaborative culture (Dean et al., 2014) and demonstrates that person-centred and professional perspectives are mutually important (Gummesson et al., 2018).

Other factors.

Sustainability of implementation emerged as a consideration in several studies (Deutschlander et al., 2012; Grant et al., 2011; VanKuiken et al., 2016) whether related to lack of administrative infrastructure (C Evans, H et al., 2011) or the absence of additional funding (Kaplan et al., 2015) and in the case of the latter, that replicability of IPE implementation is also limited (Reis et al., 2015). Identification, engagement (Packard et al., 2018) and alignment (Grymonpre, 2016) of supportive stakeholders in implementation activities (Shaw-Battista et al., 2015) was also viewed as critical to sustainability.

Key lessons—*Other factors*.

- Organisational change theory and diffusion of innovation theory should be employed as part of implementation (Packard et al., 2018).
- The use of a framework can help illustrate the changes required both within and between the educational and practice domains at micro, meso and macro levels (Grymonpre, 2016).
- Achieving harmonisation between all stakeholders is important to achieve scalable and sustainable program implementation (Grymonpre, 2016).

## Discussion

This review sought to identify the methodological characteristics, implementation components of primary studies of IPE, and the challenges and key lessons for faculty to consider when implementing IPE. Twenty-seven primary studies met the criteria for inclusion in this review. The included studies were predominantly from North America, Canada and the UK which is aligned with locations of international IPE leaders and practitioners as well as scholarship and activity in IPE.

In response to the first research question, *what are the methodological characteristics and implementation components reported in the primary IPE studies?*, the review found that study designs were mostly descriptive, case reports and the preponderance of this level of evidence is broadly in keeping with the popularity of this method in education research (Grauer, 2012). Some studies may have been conducted as pilot projects (although not necessarily identified as such) for IPE initiatives with the intention of scaling up if successful (Burns & Schuller, 2007). The reliance on case reports may also reflect educational research more broadly, which typically has low levels of investment and a deficit of experimental designs (Burns & Schuller, 2007).

Aligned with the purposes of this review, case reports may be more likely to contain rich description (Kyburz-Graber, 2004) of the implementation components of IPE activities. However, the components of implementation were variously and inconsistently reported across all included studies. No one study reported all the implementation components and in particular timing and teaching components of implementation were infrequently reported. However, all studies reported the curriculum level of IPE implementation which is essential to interpretation given that the implications for the degree of organisational change required varies according to the level of delivery (The Interprofessional Curriculum Renewal Consortium Australia, 2014).

In terms of implementation components, IPE was most commonly integrated into curricula, was optional, involved group learning, and used combinations of interactive and didactic approaches. Integration has been associated with higher-level educational outcomes (Prast et al., 2016), however, guidance on methods for effective integration is sparse (Thistlethwaite & Moran, 2010). The finding that integration was more frequent in the reviewed studies differs from those of an Australian national survey in which the majority of IPE activities were discrete (The Interprofessional Curriculum Renewal Consortium Australia, 2014).

Most of the included studies implemented optional activities and this may influence learner perceptions that these are not as important as mandatory experiences, and result in reduced learner engagement (Reeves, 2012; The Interprofessional Curriculum Renewal Consortium Australia, 2014). Optional activities can provide positive learning experiences but this implementation is associated with lower uptake (The Interprofessional Curriculum Renewal Consortium Australia, 2014). Whereas implementing a combination of both mandatory and optional learning activities, as was evident in three of the included studies, has the potential to result in perceived high status of the IPE program, offer flexibility in scheduling extra-curricular activities (Reeves, 2012), and provide interested learners with leadership development opportunities.

Group or collective learning (The Interprofessional Curriculum Renewal Consortium Australia, 2014) was most commonly reported followed by both group and individual learning. This reflects the use of small group processes in IPE and highlights the need to address issues related to group balance, size and stability (Oandasan & Reeves, 2005). Interactive learning or a combination of interactive and didactic learning were most common in the included studies consistent with the assertion that effective IPE generally utilises interactive learning in small groups with didactic methods used sparingly (The Interprofessional Curriculum Renewal Consortium Australia, 2014).

None of the studies reported whether IPE was explicit or implicit or whether it was implemented to highlight commonalities or make comparisons across professions, perhaps because these are more nuanced approaches in implementing IPE. However, because implicit IPE occurs in an unplanned, uncontrolled, and unpredictable fashion (The Interprofessional Curriculum Renewal Consortium Australia, 2014), the purposeful implementation of IPE with explicit learning outcomes is important to report. As the primary goal of IPE is to teach collaborative practice (Oandasan & Reeves, 2005), what is taught should include both commonalities, i.e. collaborative competencies, as well as comparisons, i.e. recognition of one’s own and others’ roles.

The implementation of IPE varied in duration and the length of the activity had significant impact on shaping the experience and resource implications (The Interprofessional Curriculum Renewal Consortium Australia, 2014). IPE was most commonly implemented on campus. The type of setting can influence the motivation of learners and whether they see the relevance of IPE to practice (Oandasan & Reeves, 2005). Ideally on-campus activities should be supported by deliberate opportunities for IPE in clinical placement (Lapkin et al., 2013) or in simulated clinical environments.

The predominant mode of delivery was face-to-face and synchronous with fewer studies implementing IPE online and asynchronously. Online mode for IPE is becoming more prevalent (Evans et al., 2019) and with the rapid transition to online learning in response to the COVID-19 pandemic it is likely that there will be even greater use of online mode to implement IPE. The most frequent issues identified in the student experience of online learning during COVID were difficulties with IT issues, variation in staff expertise in its use, inadequate academic interaction, lack of engagement, and insufficient peer interaction (Martin, 2020) all of which can threaten the integrity of IPE experiences.

Online modalities have been identified as a means of overcoming the logistical challenges in implementation of IPE, however there are also challenges in implementing IPE in an online format specifically, the logistics of coordination, time factors, expectations of those involved in online learning, and the need to incorporate social presence (Myers & O’Brien, 2015). Studies suggest that online learning in IPE can yield similar outcomes to face-to-face learning, for example, in communication skills (Lempicki & Holland, 2018) and there is some guidance about implementation in the literature (Ellaway & Masters, 2008) but there are gaps in the evidence about the efficacy of online IPE and its timing.

Group teaching models (co-teaching or team teaching) were most frequent in this review, which is unsurprising given that shared learning and teaching are integral to IPE. Whether IPE is implemented by individual, or group teaching, models may be a logistical and resource decision given that group teaching time is more intensive and costlier than individual teaching. However, co-teaching and team teaching are positively evaluated by learners and are collaborative processes, that provide the opportunity to role model collaborative behaviours (Crow & Smith, 2003) and have implications for professional socialisation and team formation (Oandasan & Reeves, 2005). Explicit reporting about the characteristics of the team and their roles in teaching may provide stronger evidence to guide implementation of group teaching.

In order to respond to the second research question, *what are the challenges and key lessons for faculty to consider when implementing IPE*? these were categorised as micro, meso and macro level factors. Professional and cultural beliefs of learners and educators were pervasive in the included studies and notably problems presented by professional culture may be the most significant barriers to overcome (Acquavita et al., 2014). Challenges identified across and in, the micro, and meso levels were consistent with the World Health Organization (WHO) mechanisms that shape IPE at the practice level (World Health Organization, 2010b). Those challenges relating to learning context were consistent with curricular mechanisms of program content, attendance, learning methods, shared objectives while contextual learning and faculty development reflected the educator mechanism of staff training. Likewise, leadership and resources (champions, institutional and managerial support) and administrative processes (logistics and scheduling) were also aligned with the WHO identified mechanisms that support IPE. Sustainability and supportive stakeholders emerged as additional themes, both of which are recognised as critical to successful implementation of IPE (World Health Organization, 2010b).

### Summary of key findings

Overall, the lack of complete and detailed reporting about implementation of IPE limits the ability to compare the efficacy of implementation approaches, the utility of studies to inform practice, to be replicated in other settings and to contribute to the advancement of IPE scholarship. However, the included studies highlighted micro, meso and macro challenges and yielded key lessons to guide faculty in the implementation of IPE.

### Strengths and limitations

The findings of this review should be considered in light of strengths and potential limitations. This review has focused on implementation of IPE as differentiated from design, assessment and evaluation of IPE. Although we acknowledge that these four domains are interconnected, the scope of this review means that broader assertions cannot be made about whether implementation components are effective or deliver demonstrable benefit to learners.

Although scoping reviews are not required to be comprehensive, our approach demonstrates procedural and methodological rigour. The systematic search was developed in conjunction with a Senior Librarian, with independent screening at title and abstract before paired multi professional teams assessed the eligibility of included studies. Despite this, it is possible that relevant studies have been missed. Because we did not screen papers based on the inclusion of design, implementation, assessment and/or evaluation in the title or abstract our review resulted in more comprehensive approach to the inclusion of papers than is typical in a review.

For the purposes of this review, publication in peer reviewed journals was a proxy for quality and we did not conduct quality assessment on the included studies or exclude studies based on quality. This was important in order to elucidate the methodological characteristics and extent to which implementation components are reported in peer reviewed publications. Identification of components of implementation for data charting was guided by using the delivery components of the decision-making tree proposed by a consortium of leaders in the field (The Interprofessional Curriculum Renewal Consortium Australia, 2014). However, where components were not explicitly stated we needed to make inferences based on the information provided in the studies. Thematic analysis of the text of studies was undertaken to identify challenges and key lessons using the IPECPCP framework (D’amour & Oandasan, 2005) and additional emergent themes were not excluded. Recognising the importance of stakeholder involvement as a way to enhance the usefulness of synthesised research evidence (Pollock et al., 2018) the wider project, of which this review forms a component, has been informed by an international panel of experts in interprofessional education.

### Future research

The findings of this review suggest that further research could inform a structured approach for reporting of implementation of IPE studies. This review provides guidance for faculty in implementation of IPE, further research could validate these key lessons. While beyond the scope of this review, the examination of implementation outcomes i.e. acceptability, adoption, appropriateness, feasibility, fidelity, implementation cost, penetration and sustainability (Lengnick-Hall et al., 2022) warrants further examination in relation to the implementation of IPE.

## Conclusions

This scoping review has responded to the deficit of quality methodological studies and staff resources for IPE implementation. Summarising the evidence for implementation IPE has highlighted the lack of complete and detailed reporting for implementation of IPE. The challenges in implementation of IPE should not be underestimated. Raising awareness of these and providing guidance to faculty through key lessons may contribute to improving IPE implementation quality and the level of success of IPE programs.
